# *Notes from the Field*: Typhoid Fever Outbreak — Harare, Zimbabwe, October 2016–March 2017

**DOI:** 10.15585/mmwr.mm6711a7

**Published:** 2018-03-23

**Authors:** William W. Davis, Prosper Chonzi, Kudzai P.E. Masunda, Lindsey M. Shields, Innocent Mukeredzi, Portia Manangazira, Emmaculate Govore, Rachael D. Aubert, Haley Martin, Elizabeth Gonese, John B. Ochieng, Bonaventure Juma, Hammad Ali, Kristi Allen, Beth A. Tippett Barr, Eric Mintz, Grace D. Appiah

**Affiliations:** ^1^Epidemic Intelligence Service, CDC; ^2^Harare City Health Department, Harare, Zimbabwe; ^3^Ministry of Health and Child Care, Harare, Zimbabwe; ^4^Division of Foodborne, Waterborne and Environmental Diseases, National Center for Emerging and Infectious Diseases, CDC; ^5^Division of Global HIV and TB, Center for Global Health, CDC; ^6^Kenya Medical Research Institute, Kisumu, Kenya; ^7^Division of Global Health Protection, Center for Global Health, CDC.

In October 2016, the Harare City Health Department (HCHD) surveillance system recorded the beginning of an upward trend in typhoid cases. On December 27, 2016, after the typhoid fever–associated death of a student, the Ministry of Health and Child Care (MOHCC) in Zimbabwe declared an outbreak of typhoid fever. HCHD defined a suspected case in a resident of Harare City as an illness that began on or after October 6, 2016, with fever ≥100.4°F (38°C), body pains, headache, and abdominal pain. Patients with confirmed cases had blood or stool specimens positive for *Salmonella* Typhi.

HCHD reported 860 cases with illness onset from October 6, 2016, through March 8, 2017, including 780 suspected cases, 80 confirmed cases, and four deaths (case fatality rate = 0.5%) (Figure). A spike in suspected cases on January 1 followed widespread media reports of the death of the student, but none of these cases were confirmed by lab testing. A total of 665 (77%) cases occurred in the high-density suburbs of Budiriro, Glen View, and Mbare; 24 (3%) patients were from outside Harare. Patients ranged in age from 1 month to 78 years (median age = 18 years); 48% were female.

**FIGURE:**
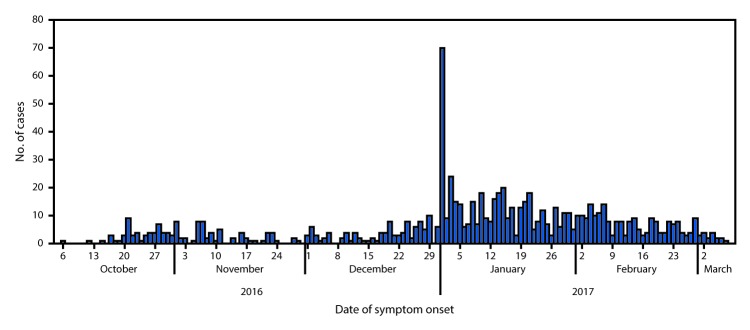
Suspected cases of typhoid fever (N = 860), by date of symptom onset — Harare, Zimbabwe, October 6, 2016–March 8, 2017

Harare’s Beatrice Road Infectious Disease Hospital tested isolates from blood and stool of 73 patients for antimicrobial susceptibility using the disk diffusion method. According to Clinical and Laboratory Standards Institute interpretive criteria (*1*), 45 (61%) were susceptible to ciprofloxacin, 10 (14%) indicated decreased ciprofloxacin susceptibility, and 18 (25%) were resistant. All but one of the 18 ciprofloxacin-resistant isolates were from patients who became ill after December 31, 2016, representing 39% of the 44 isolates from December 31, 2016, to March 8, 2017.

Assessments of affected suburbs identified 120 broken sewer lines and overcrowded apartment blocks with limited access to sanitary facilities. The area experienced frequent municipal water shortages because of an ongoing drought, and residents regularly relied on boreholes and shallow wells for drinking water (*2*). Of 32 boreholes in Mbare suburb, 18 (56%) were tested; 13 (72%) of those were contaminated with fecal coliform bacteria. Mapping indicated that cases were clustered around contaminated boreholes (*3*).

During January–July 2017, teams from HCHD, MOHCC, CDC, the World Health Organization (WHO), and UNICEF investigated risk factors for infection, monitored antibiotic resistance, and developed communications materials. WHO, UNICEF, and nongovernmental organizations complemented response efforts by HCHD and MOHCC by supporting interventions, including repairing boreholes and fitting them with inline chlorinators; repairing sewer lines; and distributing water purification tablets, jerry cans, buckets, and soap. The number of incident cases declined after implementation of the interventions; however, a resurgence occurred in Mbare in October 2017 (*4*). HCHD is continuing to explore options for improved risk reduction and disease control. This outbreak serves as a reminder that diseases from contaminated water are an ongoing public health concern. World Water Day, sponsored by the United Nations and observed each year on March 22, is an opportunity to commit to the responsible management of water, sanitation, and hygiene resources to help reduce waterborne disease around the world.
